# Application of cyclodextrins as second-sphere coordination ligands for gold recovery

**DOI:** 10.1038/s41467-023-36700-z

**Published:** 2023-03-09

**Authors:** Anne Ponchel, Eric Monflier

**Affiliations:** Univ. Artois, CNRS, Centrale Lille, Univ. Lille, UMR 8181, Unité de Catalyse et Chimie du Solide (UCCS), F-62300 Lens, France

**Keywords:** Environmental sciences, Environmental chemistry, Pollution remediation

## Abstract

Supramolecular chemistry based on cyclodextrin receptors as second-sphere ligands contribute to developing non-covalent materials with synergistic functionalities. Herein, we comment on a recent investigation of this concept, describing selective gold recovery through a hierarchical host-guest assembly specifically built from β-CD.

## Connecting gold to cyclodextrin through second-sphere coordination

Supramolecular chemistry consists of a unique interdisciplinary field of research, referring to the ability of matter to reorganize, exchange, or self-assembly through weak non-covalent interactions, such as van der Waals, hydrophobic or hydrogen binding, from a collection of complementary molecular components (generally termed molecular building blocks). Major discoveries and achievements of supramolecular chemistry in the history of modern science have been now recognized by two Nobel prizes in chemistry (in 1987 by D.J. Cram J.-M. Lehn and C.J. Pedersen, and in 2016 by J.-P. Sauvage and J.F. Stoddart). Among the panel of available organic building units, natural cyclodextrins (CDs, torus-shaped cyclic oligosaccharides composed of glucopyranose units) represent an interesting class of macrocyclic molecules readily produced from starch-rich renewable raw materials, with a hydrophilic internal cavity and hydrophilic external surface. The three most commonly used CDs are α, β, and γ-CD consisting of six, seven, and eight α-1,4-linked d-glucopyranose units, respectively. Their remarkable inclusion properties with organic, organometallic or inorganic guests have led to a wide range of practical applications spanning from drug delivery carriers, chemical sensors, and catalysts to sorbents for the removal of pollutants^1,2^. In the field of material science, the use of CDs as molecular connectors to design innovative materials and devices, hierarchically organized and spatially structured, represents a rapidly emerging field^3^. Thus, considerable progress has been made over the past 15 years allowing access to a plethora of hybrid architectures built from specific recognition, ranging from molecular species to infinite frameworks, such as for instance cyclodextrin–metal–organic frameworks (CD-MOF)^4^, cyclodextrin-covalent organic frameworks (CD-COF)^5^ or CD-based polymer hybrid porous materials^6^ or CD-based oxo- and metal clusters (CD-POM)^7,8^. In addition, the ability of CDs to act as second-sphere coordination ligands of transition-metal complexes constitutes another nice example of how native cyclodextrins can be used in some cases to selectively affect the solubility in water of resulting metal adducts by host-guest binding and lead to their irreversible (spontaneous) precipitation. It turns out that this concept of precipitation of encapsulated metal adducts by second-sphere coordination has emerged in recent years as a promising solution for the development of green technologies for selective metal separation. Focusing on gold separation, pioneering results have been highlighted by Stoddart et al. in 2013^9^ using α-CD and KAuBr_4_. They reported the rapid, spontaneous formation of a gold-rich precipitate in the form of needle-like crystals, simply by mixing the α-CD macrocycle and potassium tetrabromoaurate(III) in water. Structural analysis revealed that there is an almost perfect alignment of the α-CD units (forming a one-dimensional infinite channel), which is highly favorable to the propagation of second-sphere coordination adducts involving both the [AuBr_4_]^-^ anion and the [K(OH_2_)_6_]^+^ cation, and therefore allowing the selective isolation of 1:2 adducts. Such an arrangement exhibited a common feature with alternating blocks wherein the square-planar [AuBr_4_]^−^ was included into two α-CDs through their primary faces (tail-to-tail) and the octahedrally coordinated [K(OH_2_)_6_]^+^ involved their secondary faces (head-to-head). This process of co-precipitation between α-CD and KAuBr_4_ in water achieved promising results in terms of selective gold recovery (up to ∼80% at 20 °C), even carried out in the presence of competing salts of transition metals, such as K_2_PtBr_4_ and K_2_PdBr_4_, the latter two adopting also a square-planar geometry. Later, and to a lesser extent than previous findings, the same group described that, with α-CD, Au-rich precipitation could also occur with ordering by encapsulation of [AuBr_4_]^−^ by second-sphere coordination in the presence of heavy-alkalines Rb^+^ and Cs^+^ as counterions, with recovery values ranging from 41 to 68% at 20 °C^10^. Efforts were also devoted to developing an efficient gold-recovery process on a laboratory scale whose principle is simple: the co-precipitate can be collected by decanting, then chemically reduced by a reducing agent to release gold in the form of colloidal particles while the α-CD can be recycled and recrystallized before its reuse. Although the yields of recovered gold were already appealing (without the need of toxic cyanide or mercury sources), there is the perception that the process as presented is not yet versatile enough to be really implemented. Indeed, the method shows several limitations and shortcomings: (i) the supramolecular co-precipitation process requires stringent experimental conditions for optimizing the attractive stereoelectronic interactions, such as a relatively high gold concentration, limited range of acid pH (4–6) and neutralization with KOH, (ii) the use of α-CD does not constitute the perfect eco-friendly alternative for gold recovery from an industrial point of view, due to its relatively high cost, and (iii) the process cannot be extended to the more available native cyclodextrin on the market (i.e. β-CD) nor to all square-planar gold complex anions because of a less favorable molecular recognition.

## A further step toward higher sustainability?

Few scientific research follows a linear path, but nevertheless, the results above described have great resonance in the succeeding work. Ten years after their first work on gold recovery, Stoddard et al.^11^ achieve a new important step forward to recovering gold from waste, with almost 100% efficiency, by developing an additive-induced supramolecular co-precipitation process based on the use of β-CD as a second-sphere ligand. The exploitation of the concept combining specific molecular recognition, metal coordination-driven infinite self-assembly from β-CD, instead of α-CD, constitutes a straightforward strategy for low-cost and eco-friendly synthetic protocols. Indeed, β-CD is the most frequently used cyclodextrin derivative as it is more cost-effective and available as compared to other cyclodextrins. β-CD is also much less water-soluble (18.5 g/L against 145 g/L for α-CD), which makes it more desirable for future development in crystal growth technologies. Moreover, its molecular structure allows in principle the formation of inclusion complexes with a wide variety of organic guests of different shapes and polarities, in accordance with the most extensive molecular recognition data available in the literature^12^. More specifically, the authors report on a three-component supramolecular hybrid co-precipitate, built from selective recognition involving the β-CD, the anionic square-planar Au(III) complex ([AuBr_4_]^−^) and a hydrophobic organic molecule. The ability to interact is carefully investigated by studying specific interactions between β-CD and both types of guest units through a bottom-up approach, carried out both in solution and in solid-state. Notably, β-CD shows a remarkable affinity for the metal complex in the form of a 1:1 host–guest anionic complex, making its use as a hybrid building block possible. It is shown that β-CD preferentially interacts with the [AuBr_4_]^−^ moiety through the primary face of the molecular receptor, again functioning as a second-sphere coordination ligand. However, no rapid precipitation is observed with this two-component mixture. To overcome this, Stoddart and coworkers elegantly demonstrate that the small addition of a third component (typically a hydrophobic solvent molecule) to this preassembled KAuBr_4_⊂β-CD adduct is sufficient to initiate fast and quantitative solid-state polymerization. Among the twelve different additives examined, dibutyl carbitol (DBC) results in maximum yields of gold recovery (close to 100%), even in volume fractions as low as 0.1 % (v:v). Satisfactory results are also obtained with two other fairly common organic molecules such as toluene (∼95%) and chloroform (∼80%) whereas no gold recovery is initiated using triglycerides (oils) or low sterically hindered small molecules (diethyl ether or ethyl acetate). The scope of the additive-induced method seems to be limited to classes of organic compounds, which can be included in natural β-CD and which also show favorable physical properties (low vapor pressure, high flash point). In line with what has been observed in the studies with α-CD, the DBC-induced co-precipitation process is also shown to be highly selective for gold, remaining completely ineffective in the case of Pt and Pd counterparts. From a structural point of view, single-crystal X-ray diffraction reveals a one-dimensional infinite sandwich-type structure wherein DBC alternates with the ditopic supramolecular unit [HAuBr_4_⊂2β-CD] through the secondary faces of the β-CD, adopting a confined folded conformation. Ultimately, a linear infinite supramolecular polymer propagates along the *c*-axis (See Figure 3c in Ref. ^11^) and stacks together to give needle-like crystals. The efficacy of this methodology based on additive-induced supramolecular co-precipitation from gold is also demonstrated in more realistic conditions from an industrial point of view. For example, high gold recovery yields (always above 90%) are obtained, regardless of the Au concentration used (even for values as small as several ppm that are well-representative of real industrial leaching solutions). The co-precipitation process also exhibits an excellent tolerance to all types of cations (H^+^, K^+^, or Na^+^) associated with the anionic gold complex, meaning that the KOH addition is no longer necessary in the process. Perhaps finally, the most promising aspect of the study is the highlighting of the applicability of the gold recovery process from real spent electronic scraps. The protocol developed by the authors consists of several steps (etching in HBr-H_2_O_2_, pH adjusting, filtering insoluble impurities) before the addition of the β-CD-DBC mixture. Notably, despite the presence of residual metal impurities in the leached solution, yellow crystallites are easily formed and isolated, and their characterization is perfectly in line with the expected [HAuBr_4_∙DBC⊂2β-CD] structure.

## Outlook

In light of the above results, it appeared that supramolecular science, especially based on renewable natural cyclodextrins, reached a level of maturity that enables a paradigm shift. Its progress has concomitantly occurred with the development of synthetic, analytical, and characterization methods that have enabled the discovery of hierarchically ordered materials on relevant scales. In context, the second-sphere coordination has evolved as a strategic field research for the elaboration of hybrid superstructures for which an exciting future is foreseen. The recent work of Stoddart and colleagues has illustrated in a rational and elegant manner the design of a class of metal-organic superstructures, built from different types of well-defined components, and formed by the participation of cooperative multi-recognition and infinite self-assembly (from the solution to the solid-sate). This consortium brought evidence of the outstanding selective gold extraction application carried out under benign conditions. There is considerable interest to develop more eco-friendly processes that can selectively recover and reuse gold metal resources originating from polycontaminated wastes generated by various industries (electric wastes, electronic equipment, jewelry, anode slimes, hydrometallurgy). Their construction based on the non-covalent binding of β-CD and metal complexes opens the way for innovative and disrupting devices in technologically-important fields. Nevertheless, the second-sphere coordination concept still has a lot to unveil. One of the future challenges could be the development of new CD-based protocols adapted to the recovery, separation, and purification of other heavy metals, including those contained in radioactive wastes or in spent catalysts. Considering however, the large structural diversity of available guests, offering species that differ in their composition, shape, size, symmetry, and ionic charge, there should have extensive and in-depth knowledge of these macrocyclic supramolecular assemblies to determine the factor dictating their supramolecular interplay with metals of interest.
